# Norovirus GII.17 Natural Infections in Rhesus Monkeys, China 

**DOI:** 10.3201/eid2302.161077

**Published:** 2017-02

**Authors:** Zhanlong He, Bo Liu, Yufen Tao, Chao Li, Ming Xia, Weiming Zhong, Xi Jiang, Hongqi Liu, Ming Tan

**Affiliations:** Institute of Medical Biology at the Chinese Academy of Medical Science, Kunming, Yunnan Province, China (Z. He, B. Liu, Y. Tao, C. Li, H. Liu);; Cincinnati Children’s Hospital Medical Center, Cincinnati, Ohio, USA (M. Xia, W. Zhong, X. Jiang, M. Tan)

**Keywords:** Norovirus, monkey, cross-species transmission, histo-blood group antigens, natural infection, viruses, China, enteric infections, *Suggested citation for this article*: He Z, Liu B, Tao Y, Li C, Xia M, Zhong W, et al. Norovirus GII.17 natural infections in rhesus monkeys, China. Emerg Infect Dis. 2017 Feb [*date cited*]. http://dx.doi.org/10.3201/eid2302.161077

## Abstract

Noroviruses are a leading viral cause of acute gastroenteritis among humans. During the 2014–15 epidemic season, norovirus GII.17 was detected in rhesus monkeys in China. Genetic, structural, and challenge studies revealed virus mutations and verified the infections. Thus, cross-species transmission may occur, and monkeys may be a virus reservoir.

Noroviruses are a leading viral cause of epidemic and sporadic acute gastroenteritis in humans of all ages, causing substantial illness and death. Each year, noroviruses cause ≈21 million infections in the United States and ≈200,000 deaths worldwide. Among the 6 known norovirus genogroups (GI–VI), all GI, most GII, and a few GIV noroviruses infect humans (human noroviruses). Each genogroup includes up to 22 genotypes based on the sequences of major capsid protein 1 (VP1). Although GII.4 noroviruses were predominant globally for 2 decades, the previously rare GII.17 genotype emerged during the 2014–15 epidemic season in China and other Southeast Asian countries/regions, causing major epidemics of acute gastroenteritis (*1*,*2*). Infection of domestic pigs, cattle, dogs, and rhesus macaques with human norovirus has been reported (*3*–*7*). We report the detection and characterization of norovirus GII.17 that extensively and naturally infected farm-raised rhesus monkeys in southwestern China.

## The Study

In January 2015, a total of 50 fecal samples were randomly collected from the general monkey population at a farm with ≈2,000 monkeys in Kunming City, Yunnan Province, China, in accordance with the guidelines for humane treatment of animals and approved by the Institutional Animal Care and Use Committee of the Institute of Medical Biology at the Chinese Academy of Medical Science. Viral RNA was extracted from 10% fecal suspensions in physiologic saline by use of a QIAGEN Mini RNA kit (Hilden, Germany). We randomly selected 28 RNA samples for calicivirus detection with a 1-step reverse transcription PCR that used the primer pair P289 and P290 (*8*), designed to amplify a genome fragment encoding the calicivirus RNA-dependent RNA polymerase. One of the samples showed the expected 310-bp calicivirus RNA-dependent RNA polymerase gene fragment, which was confirmed by DNA sequencing. Nucleotide BLAST (http://blast.ncbi.nlm.nih.gov/Blast.cgi) analysis indicated that this gene fragment was from a GII.17 norovirus, which we named Mk/KM1509/Yunnan/CHN/2015 (monkey GII.17 norovirus).

**Figure 1:**
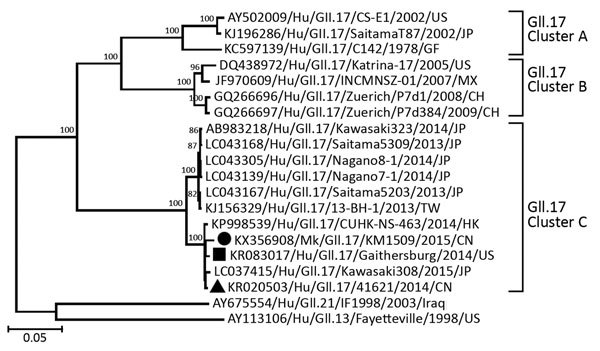
Phylogenetic analysis based on the viral capsid protein 1 genes of the monkey GII.17 norovirus and other reference human GII.17 noroviruses. The analysis involved 20 full-length viral capsid protein 1–encoding genes (gene identification shown), including 17 previously reported GII.17 human norovirus representatives. Black square indicates noroviruses reported in (*9*); black triangle indicates human GII.17 variants circulating in China as reported in (2); black circle indicates the monkey GII.17 norovirus from this study. Comparison viruses are 1 from GII.13 genotype and 1 from GII.21 genotype. The evolutionary history was inferred by using the neighbor-joining method. The optimal tree with the branch length sum of 0.91354301 is shown. The percentage of replicate trees in which the associated taxa clustered together in the bootstrap test (1,000 replicates) are shown above the branches. The tree is drawn to scale; branch lengths are in the same units as those of the evolutionary distances used to infer the phylogenetic tree. The evolutionary distances were computed by using the Tajima-Nei method and represent the number of base substitutions per site. The analysis involved 20 nt sequences. All positions containing gaps and missing data were eliminated. The final dataset contained a total of 1,265 positions. Evolutionary analyses were conducted in MEGA6 (http://www.megasoftware.net).

Next, we amplified and sequenced the full ≈7.5-kb genome of this norovirus. Sequence analysis showed that the monkey GII.17 norovirus genome sequence shared 99% nt identity with the human GII.17 norovirus recently detected in China (*2*). Phylogenetic analysis among representative full-length VP1-encoding genes revealed 3 clusters of GII.17 noroviruses (A, B, C) (Figure 1) (*9*). The monkey GII.17 norovirus grouped with cluster C of the recently detected GII.17 human noroviruses in China. To estimate the infection rate of monkey GII.17 norovirus in the monkey population, we designed a new pair of specific primers (199 and 200) based on our newly isolated genome sequence to reanalyze the 50 extracted RNA samples. PCR amplification and DNA sequencing of the PCR products indicated identical GII.17 noroviruses in 16 (32%) samples. 

**Figure 2:**
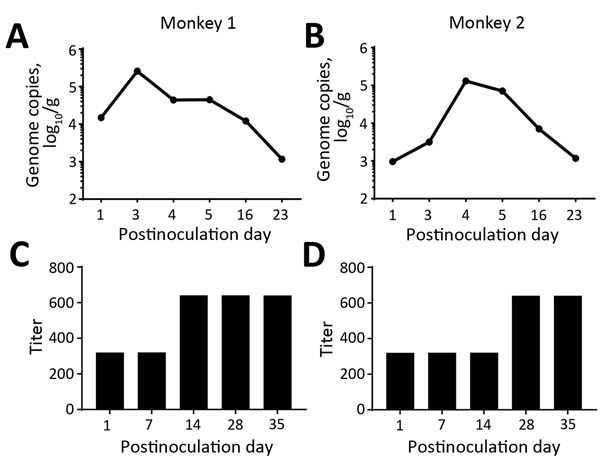
Challenge testing of 2 norovirus-negative macaques with GII.17 norovirus. A, B) Challenged macaques shed norovirus-specific RNA (genome copies) for at least 16 days; shedding peaked on postinoculation day 3. C, D) Serum norovirus antibody titers before (postinoculation day 1) and after (postinoculation day 7–35) challenge.

We then performed a challenge experiment to assess infection and replication of this GII.17 norovirus in monkeys. We randomly selected 2 monkeys for which fecal samples were negative for norovirus and intragastrically administered (by nasogastric tube) a GII.17-positive fecal sample (consisting of 1 mL filtered 20% fecal suspension containing 8.3 × 10^5^ norovirus genome copies). Despite the absence of typical signs (watery diarrhea and fever), both challenged animals shed norovirus RNA in their feces for at least 16 days; by postinoculation day 3, shedding peaks were 2.573 × 10^5^ genome copies/gram feces for 1 monkey and 1.33 × 10^5^ for the other (Figure 2, panels A, B). These great increases of the shed genome copies indicated successful infection and replication of the GII.17 norovirus in monkeys.

We also measured possible seroconversion in the challenged animals by using recombinant VP1 protein of the monkey GII.17 norovirus developed after the challenge was performed. ELISA with monkey norovirus VP1 as the capture antigen showed that both monkeys had high norovirus IgG titers (1:320) before the challenge. As a result, norovirus-specific antibody titer increases for both challenged animals were only 2-fold (Figure 2, panels C, D). The observed high preexisting norovirus antibody titers in both monkeys selected for challenge may have resulted from previous infection with the GII.17 norovirus, although their fecal samples were norovirus negative by the time of selection for challenge. The observed low antibody responses and the lack of typical clinical signs after norovirus infection via virus challenge may result from relatively high preexisting GII.17 antibody titers. Further study to define the role of preexisting norovirus antibodies in norovirus infection of rhesus monkeys is needed.

Histo-blood group antigens (HBGAs) are norovirus host factors in which hosts with matched HBGA types exhibit increased susceptibility to norovirus infection (*10*). To improve understanding of the HBGA binding profile of this monkey GII.17 norovirus, the recombinant VP1 proteins of the new monkey strain and a recent human GII.17 norovirus were expressed in *Escherichia coli* (Technical Appendix Figure, panel A), as previously described (*11*). HBGA binding assays, performed by using defined human saliva samples with known HBGA types, revealed that the VP1 protein of the monkey GII.17 norovirus bound to human saliva samples with significantly lower binding signals (optical densities) than the human GII.17 norovirus (Technical Appendix Figure, panels B and C). Accordingly, sequence comparisons of the P domain (the HBGA binding domain) between the human and monkey noroviruses and structural analysis based on the known GII.17 P dimer crystal structure (*12*) revealed 2 residue mutations, D377G and N342S, near the HBGA binding site of the monkey GII.17 norovirus (Technical Appendix Figure, panel D). The D377G mutation of the monkey GII.17 norovirus replaces the negatively charged aspartic acid with a small, neutral glycine; the N342S mutation replaces the larger, strongly polar asparagine with a tiny, weakly polar serine. These 2 mutations may be the reason why binding of the monkey GII.17 norovirus to HBGAs is weaker than that of the GII.17 human norovirus. We also noted that the monkey GII.17 VP1 protein bound saliva samples with significantly higher binding signals to saliva samples of type B, which also happens to be the major blood type of rhesus monkeys (*13*).

## Conclusions

Although limited success during monkey challenge studies using human noroviruses has been reported (*14**,**15*), our study showed that GII.17 noroviruses were able to infect a monkey population, indicating extensive human norovirus infection of farm-raised rhesus monkeys under natural conditions. Our findings suggest that it may be possible to establish a useful animal model of norovirus infection to evaluate human norovirus vaccines and antiviral drugs and to study human norovirus pathogenesis, although further testing needs to be done to confirm such possibility. Our findings also raise new concerns about possible viral reservoirs and cross-species transmission of noroviruses.

Considering the fact that a new GII.17 variant emerged as the predominant norovirus and caused major epidemics in China during the same period (*1*,*2*), the detected monkey GII.17 norovirus probably originated from a human GII.17 norovirus. However, the mutations near the HBGA binding site might imply an initial adaptation of the monkey GII.17 norovirus to the new host. To provide a better understanding of its infection, pathogenesis, host specificity, epidemiology, and cross-species transmission, further characterization of this monkey GII.17 norovirus is warranted. This information may also be valuable for the future establishment of a monkey model of norovirus infection for vaccine and antiviral evaluation and for addressing the concerns of unknown viral reservoirs and potential zoonotic infection of noroviruses.

Technical Appendix Production and characterization of the monkey and human GII.17 norovirus recombinant viral capsid protein 1.
