# Supplementary material for: Norovirus GII.17 Natural Infections in Rhesus Monkeys, China
Source: Emerg Infect Dis. 2017 Feb;23(2):316–9. doi: 10.3201/eid2302.161077 (PMC5324814; doi:10.3201/eid2302.161077)
Supplement: Technical Appendix — Production and characterization of the monkey and human GII.17 norovirus recombinant viral capsid protein 1. [file 16-1077-Techapp-s1.pdf]

Article DOI: http://dx.doi.org/10.3201/eid2302.161077

Norovirus GII.17 Natural Infections in Rhesus Monkeys, China

Technical Appendix

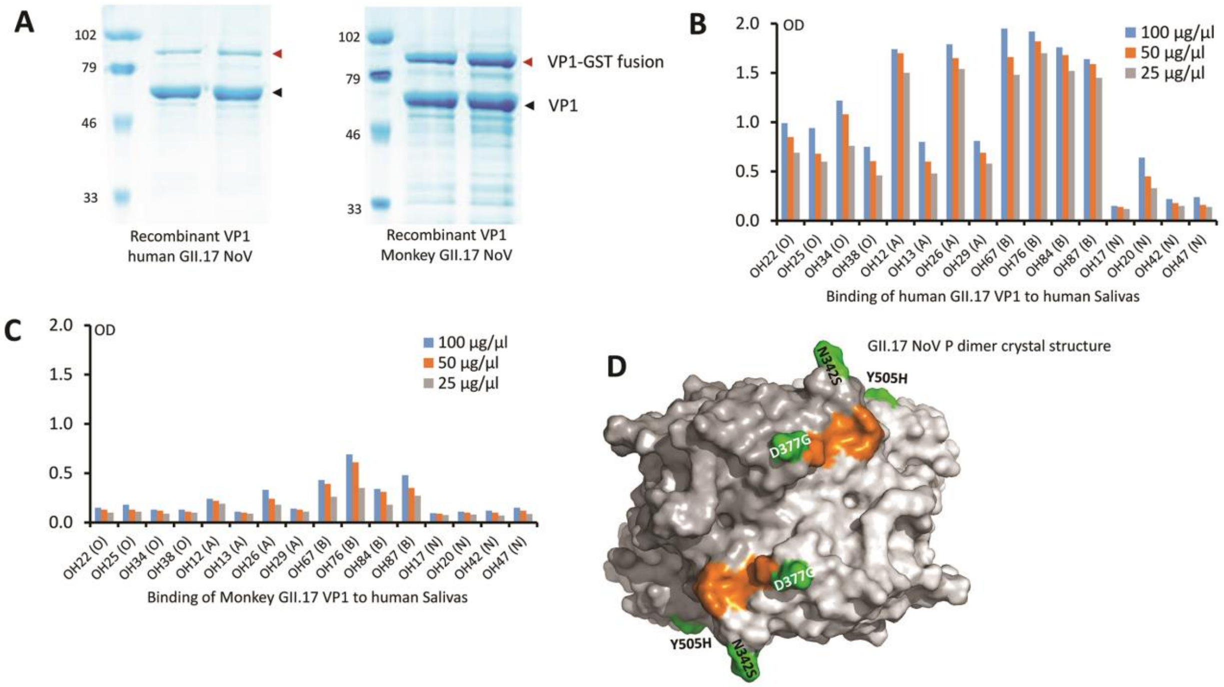

Technical Appendix Figure. Production and characterization of the monkey and human GII.17 norovirus (NoV) recombinant viral capsid protein (VP) 1. A) Expression of the human (left) and monkey (right) GII.17 NoV recombinant VP1 proteins. The human GII.17 NoV was the most recent variant (DG42) isolated from a NoV outbreak (*1*). B and C) The binding signal intensities (optical densities [ODs], y-axis) of human (B) and monkey (C) GII.17 VP1s to the same set of saliva samples (x-axis), with defined histo-blood group antigen (HBGA) types, through saliva-based HBGA binding assays. The human saliva samples were collected from healthy volunteers in Ohio, USA, with defined type O, A, B, and nonsecretor (N) types. VP1 proteins at 3 indicated concentrations were used for the assays. D) The GII.17 P dimer

crystal structure (PDB code 5F4O). The HBGA binding sites are shown in orange; 3 surface mutations (N342S, D377G, and Y505H) are shown in green and amino acids are indicated.

Reference

1. Zhang XF, Huang Q, Long Y, Jiang X, Zhang T, Tan M, et al. An outbreak caused by GII.17 norovirus with a wide spectrum of HBGA-associated susceptibility. Sci Rep. 2015;5:17687. PubMed http://dx.doi.org/10.1038/srep17687
